# Effect of Letrozole on sperm parameters, chromatin status and ROS level in idiopathic Oligo/Astheno/Teratozoospermia

**DOI:** 10.1186/s12958-020-00591-2

**Published:** 2020-05-13

**Authors:** Leila Kooshesh, Soghra Bahmanpour, Shahriar Zeighami, Mohammad Hussain Nasr-Esfahani

**Affiliations:** 1 Department of Anatomy, Shiraz University of Medical Sciences, Shiraz, Iran; 2 Department of Urology, Shiraz University of Medical Sciences, Shiraz, Iran; 3 Department of Reproductive Biotechnology, Reproductive Biomedicine Research Centre, Royan Institute for Biotechnology, ACECR, Isfahan, Iran; 4 Isfahan Fertility and Infertility Centre, Isfahan, Iran

**Keywords:** Letrozole, Idiopathic oligo/astheno/teratozoospermia, Hormonal profile, Sperm DNA fragmentation, ROS, Protamine deficiency

## Abstract

**Background:**

This study investigates the effect of letrozole on hormone profiles, semen parameters, body mass index (BMI), degree of oxidative stress and sperm chromatin integrity in men with idiopathic oligo/astheno/teratozoospermia (iOAT) and T:E_2_ ratio ≤ 10.

**Materials and methods:**

This study is a longitudinal, prospective, interventional and open-labelled clinical trial. Semen samples were collected from 20 iOAT men with low serum testosterone (T) to estradiol (E_2_) ratio (T:E_2_ ratio ≤ 10). The participants were treated with 2.5 mg letrozole orally per day for 3 months. Then, sperm parameters, hormone profiles, BMI, chromatin integrity and intracellular reactive oxygen species (ROS) level were assessed pre- and post- treatment. The chromatin integrity was evaluated by assessment of DNA fragmentation (with TUNEL assay) and protamine deficiency (with Chromomycin A3, CMA3). Also, the intracellular ROS levels were investigated by 2′, 7′-dichlorodihydrofluorescein diacetate (DCFH-DA) staining. Finally, the differences between the parameters evaluated before and after letrozole treatment were analyzed with the t*-test* and the Wilcoxon signed-rank test.

**Results:**

Sperm concentration, percentage of sperm motility and its normal morphology increased significantly after letrozole treatment. Moreover, serum testosterone level increased but estradiol level decreased significantly following treatment. The mean of T:E2 ratio improved 1600%. Also, letrozole treatment significantly reduced the percentage of sperm TUNEL positivity and sperm CMA3 positivity. While no significant difference was observed between intracellular ROS levels and BMI before and after treatment. Finally, as a notable result, four spontaneous pregnancies (20%) were achieved after treatment.

**Conclusions:**

Letrozole treatment can effectively increase spontaneous pregnancies by improving sperm parameters and sperm chromatin integrity in men with iOAT and T:E2 ratio ≤ 10.

**Trial registration:**

Trial registration: IRCT, IRCT20191030045283N1. Registered 16 November 2019 - Retrospectively registered, https://fa.irct.ir/user/trial/43484/view

## Background

The mechanisms associated with idiopathic male infertility, mostly iOAT, are not fully known and need further investigation [1–3]. A number of factors may be involved in the genesis of this condition, including age [4], non-inflammatory alterations in the function of post testicular organs like prostate [5, 6], infective agents [7], environmental disturbances [8, 9], subtle hormonal anomalies and genetic and epigenetic factors [8]. Considering these probable etiologies, opportunities have been provided for further research in new approaches of empirical medical therapy.

Based on the understanding of hormonal control of spermatogenesis, several medications such as gonadotropins, androgens, estrogen receptor blockers (clomiphene and tamoxifen citrate), and aromatase inhibitors as empirical treatments are used for idiopathic male infertility [10–13]. The hormonal profile of men with iOAT has demonstrated lower serum testosterone level, higher serum estradiol level, and therefore, lower T:E2 ratio than normal men [8]. In addition, a research has shown that the mean of T (ng/dl):E_2_(pg/ml) in men with severe infertility and normal conditions is 6.9 and 14.5, respectively. According to this research, a cut-off point 10 was proposed as the lower limit of the T: E2 ratio for normal men [14]. Therefore, based on such observations, aromatase inhibitors (especially letrozole) have gained much attention for the treatment of iOAT with T:E_2_ ratio ≤ 10 [15–19].

Letrozole as an aromatase inhibitor has been widely used in the treatment of female infertility [20–22]. There are limited studies that have evaluated the therapeutic potential of letrozole in male infertility [15–19]. Letrozole is a selective and highly potent aromatase inhibitor that inhibits the enzyme activity of intracellular aromatase. Therefore, it can reduces the conversion of testosterone to estradiol and androstenedione to estrone [23]. Inhibiting estrogen production can reduce the negative feedback on the hypothalamus-pituitary axis, resulting in increase of FSH and LH (luteinizing hormone) levels which, in turn, can increase testosterone and stimulate spermatogenesis [23, 24].

There is a positive relationship between letrozole and improvement of sperm concentration, percentage of sperm motility and morphology [15–19]. But regarding to our information, there is no research about the effect of letrozole on sperm functional characteristics, while the sperm DNA constitute approximately 50% of the future progeny DNA, and chromatin anomalies have a negative impact on fertilization rate, embryo development, live birth and abortion rate [25–33]. Therefore, the aim of this study is investigating the effect of letrozole on hormone profiles, conventional semen analysis, BMI, the degree of oxidative stress and sperm chromatin integrity in men with iOAT and T:E2 ratio ≤ 10.

## Materials and methods

### Patients selection

This study is a longitudinal, prospective, interventional and open-labelled clinical trial (Trial Registration: IRCT20191030045283N1, Registered 16 November 2019) and has received the approval of the Ethical Board of Shiraz University of Medical Sciences (IR.SUMS.REC.1397.273). During 2018 and 2019, the patients with iOAT and a serum T:E2 ratio of ≤10 [15–19], referred to male infertility clinics affiliated with Shiraz University of Medical Sciences, were considered as candidates for inclusion in our study. Individuals with leukocytospermia (more than 1 million/ml), tobacco or alcohol abuse, ongoing medical treatment (gonadotropins, anabolic steroids, non-steroids anti-inflammatory drugs and cancer chemotherapy), previous cancer radiotherapy or chemotherapy, varicocele and Klinefelter syndrome were excluded in this study [18]. Also, the candidates that their female partners had a history of gynecological problems were excluded from the research [19]. A total of 20 eligible males with mean age of 34.9 years old, ranging from 24 to 43 years, were recruited in this study and all of them finished the study correctly. Also, the participants were informed about the study objectives and signed an informed consent form.

All the participants were orally treated with 2.5 mg letrozole (Soha Pharma Company, Iran) per day for 3 months [15–18]. Semen parameters, sperm chromatin integrity, intracellular ROS level and BMI was evaluated before and after letrozole administration, and also serum testosterone and estradiol levels were measured at the end of treatment.

### Semen sample

Semen samples were provided by masturbation following 3–5 days of sexual abstinence before and after 3 months of letrozole administration. A proportion of each semen sample was analyzed according to the World Health Organization guidelines (WHO, 2010) [34]. The remaining semen samples were used for the analysis of sperm DNA fragmentation by TUNEL assay, sperm protamine deficiency by CMA3 staining and sperm intracellular ROS level by DCFH staining. If the sample was not enough to carry out all these tests, a second semen sample was provided by the participant after 3–5 days of sexual abstinence.

### Assessment of sperm protamine deficiency

In this study, protamine deficiency was assessed by Chromomycin A3 (CMA3). The CMA3 competes with protamine, the main protein involved in sperm DNA packaging, for binding to small grooves and guanine and cytosine-rich regions of DNA. Therefore, the percentage of CMA3 staining correlates inversely with the protamination status, and indirectly assesses the degree of protamine deficiency [35, 36]. For this purpose, after washing the semen samples with phosphate-buffered saline (PBS) free of Ca^2+^ and Mg^2+^, the samples were fixed with carnoy’s solution (methanol: glacial acetic acid 3:1) for five minutes at 4 °C. The prepared smears were stained for 20 min with 100 μl of CMA_3_ solution (25 mg/ml CMA_3_ in a buffer called Mcllvaine [7 ml citric acid (0.1 mmol/l), 32.9 ml Na_2_HPO_4_, 7H_2_O (0.2 mmol/l), PH 7.0 containing 10 μmol/l MgCl_2_)]). Subsequently, each smear was rinsed in the same buffer and mounted with buffered glycerol [1]. Olympus fluorescence microscope (BX61, Tokyo, Japan) with the appropriate filters (460–470 nm) was used for analysis. For each participant, 200 spermatozoa were assessed. The CMA3 positive sperm was distinguished from the CMA3 negative one through its bright yellow staining [35].

### Assessment of sperm DNA fragmentation

In our study, for assessment of sperm DNA fragmentation, terminal deoxynucleotidyl transferase-mediated fluorescein-dUTP nick end labeling (TUNEL) assay was carried out by Promega DeadEnd™ Fluorometric TUNEL kit. In this assay, single and double DNA strand breaks at the 3’OH ends are labeled by fluorescent dUTP nucleotides using the template and primer independent TdT (terminal deoxynucleotidyl transferase) and then fluorescence is detected by flow cytometry [37]. In this study according to the kit manufacturer instructions, a minimum of 3 × 10^6^ sperm cells from each semen sample were washed twice in PBS and fixed by 8% paraformaldehyde for 25 min at room temperature. The cells were then Permeabilized by 0.4% Triton X-100 solution and equilibrated with equilibration Buffer for 7 min at room temperature. After washing, the cells were incubated for 60 min at 37 °C in the DNA-labeling solution (including Nucleotide Mix and TdT). Along with the test sample, analysis was also done for negative control sample. The negative control sample was prepared by DNA-labeling solution without TdT enzyme. Finally, test and negative control samples were washed twice in PBS and diluted to a final volume of 300 μl in PBS.

After preparation of samples, the fluorescent was detected by flow cytometer (FACS calibur flow cytometer; Becton Dickinson san Jose CA, USA) and the obtained data were then analyzed using the flowjo software (Version 7.6).

### Assessment of sperm intracellular ROS level

Intracellular ROS level was measured using a flow-cytometry based DCFH-DA staining. DCFH-DA, a non-fluorescent probe, can pass the cell membrane. In the cell, DCFH-DA is deacetylated to DCFH by cellular esterase. DCFH is another non-fluorescent probe that cannot cross the cell membrane. DCFH reacts with H_2_O_2_ (an intracellular ROS) to form DCF that is a fluorescent probe and can be detected by flow cytometry [38, 39].

For this assay, a sperm suspension (including 2 × 10^6^ sperm/ml in PBS) was exposed to 0.5 μm DCFH- DA and incubated at 37 °C for 30 min. Along with the test sample, a negative control sample was prepared only by PBS. Samples were then washed twice in PBS. Finally, the samples were diluted to a final volume of 400 μl PBS and analyzed by flow cytometry [38, 40]. Flow cytometry data were then analyzed using the flowjo software (Version 7.6).

### Statistical analysis

Statistical analysis was performed by the prism and SPSS software. The population normality was verified with the D’Agostino-Pearson omnibus test. The differences between the assessed parameters before and after letrozole treatment were evaluated with the *t-test* for the paired samples or the Wilcoxon signed-rank test, depending on the distribution of the parameters. Associations among quantitative parameters were analyzed by the Spearman correlation coefficient. Results are presented as mean ± SEM (Standard Error of Mean). Probability value of less than 0.05 was considered to be statistically significant.

## Results

In this study, 20 participants with iOAT and serum T:E_2_ ratio ≤ 10 were treated with letrozole for 3 months. These infertile men were evaluated for semen parameters, hormone profiles, BMI, sperm DNA fragmentation, protamine deficiency and sperm intracellular ROS level pre-and post-treatment. No severe side-effects were reported during the treatment, only five patients declared the reduction of their libido. Also, following letrozole administration, four spontaneous pregnancies (20%) were reported.

### Effect of letrozole on hormone profiles, semen parameters and BMI

**Fig. 1:**
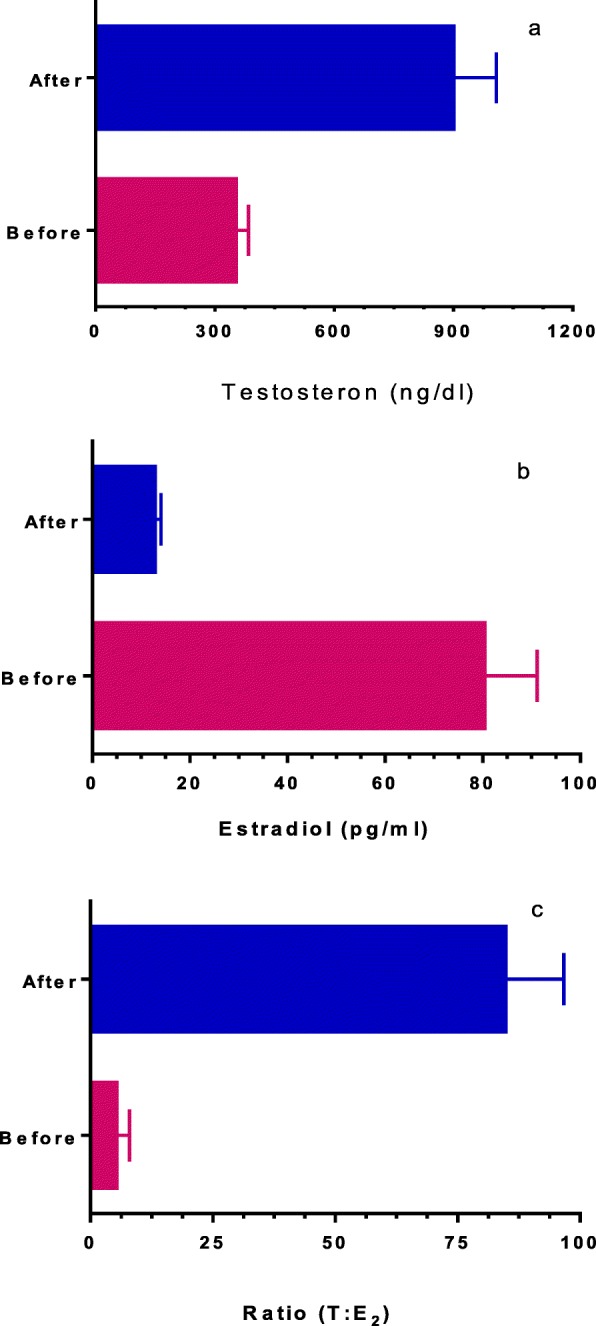
Comparison the mean hormone profiles before and after letrozole treatment: **a**) Serum Testosterone level (*P* = 0.0001), **b**) Serum estradiol level (*P* < 0.0001) and **c**) Ratio (T: E_2_, *P* < 0.0001)

Figure 1a-c compare hormone profiles before and after 3 months of letrozole administration. The results of Fig. 1a-b show that the mean of serum testosterone level (ng/dl) increased significantly (351.7 ± 32.85 versus 899.3 ± 108.7; *P* = 0.0001), and conversely, the mean of serum estradiol level (pg/ml) decreased significantly (80.29 ± 10.9 versus 12.79 ± 1.31; *P* < 0.0001) after the administration of letrozole. In all patients, the T:E_2_ ratio increased significantly (5.23 ± 0.61 versus 84.69 ± 12.03; *P* < 0.0001) after 3 months of letrozole treatment (Fig. 1c).

**Fig. 2:**
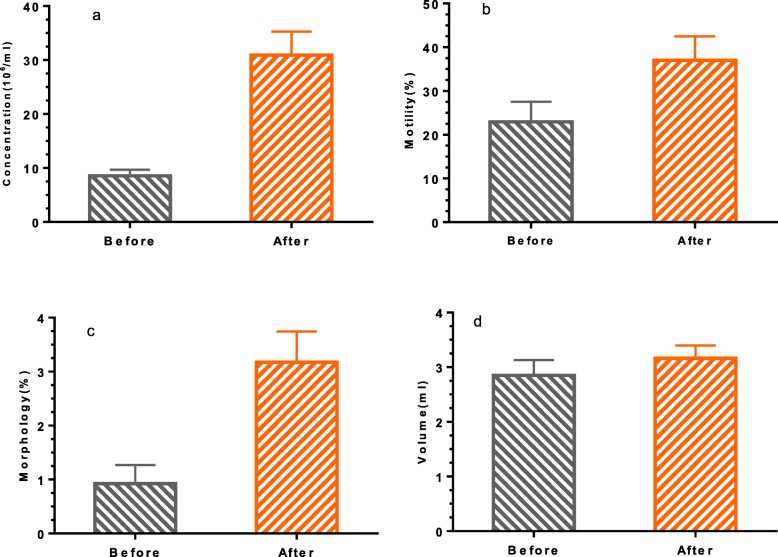
Comparison the mean semen parameters before and after letrozole treatment: **a**) Sperm concentration (*P* < 0.0001), **b**) Sperm motility% (*P* = 0.035), **c**) Sperm normal morphology% (*P* < 0.0001) and **d**) Volume (*P* = 0.299)

Figures 2a-d compare sperm parameters before and after 3 months of letrozole administration. The mean of sperm concentration (10^6^/ml) increased significantly (8.565 ± 1.13 versus 30.93 ± 4.36; *P* < 0.0001) following treatment (Fig. 2a). The mean of sperm motility percentage increased significantly (22.95 ± 4.63 versus 37 ± 5.48; *P* = 0.035) after letrozole treatment (Fig. 2b). Also, the mean of normal sperm morphology percentage increased significantly (0.925 ± 0.34 versus 3.175 ± 0.57; *P* < 0.0001) following letrozole administration (Fig. 2c). There is no significant difference between the mean ejaculation volume (ml), (2.85 ± 0.27 versus 3.16 ± 0.23; *P* = 0.299), before and after letrozole treatment (Fig. 2d).

There is also no significant difference between the mean BMI (kg/m^2^) from pre-treatment to post-treatment (29.81 ± 3.63 versus 30.04 ± 3.52). Moreover, no significant correlation was observed between the baseline BMI and improvement of sperm parameters, hormonal balance, chromatin status and the intracellular ROS level following the administration of letrozole.

### Effect of letrozole on the sperm chromatin status and the intracellular ROS level

In this study, in order to assess the chromatin status, sperm DNA fragmentation and protamine deficiency were evaluated by TUNEL and CMA3 staining, respectively. Further, the intracellular ROS levels were assessed by DCFH-DA staining.

**Fig. 3:**
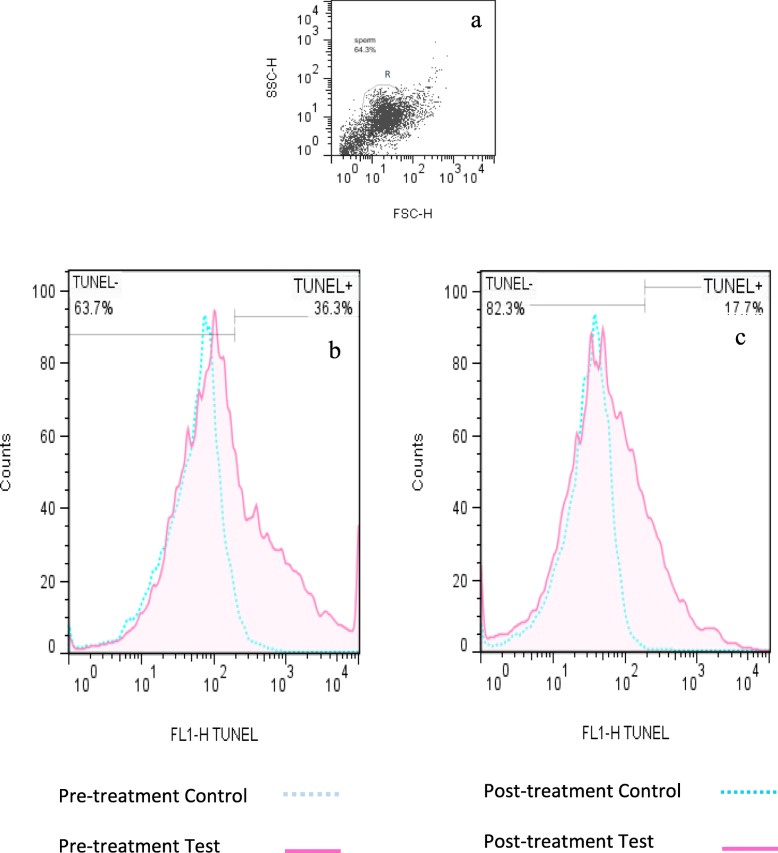
Comparison of flow cytometry analysis of TUNEL positive% spermatozoa obtained from iOAT men with T:E_2_ ratio ≤ 10, before and after letrozole treatment. **a**) Dot plot of spermatozoa. The cells gated in R1 region were analyzed; debris was excluded from the analysis. **b**) Pre-treatment TUNEL positivity% in test semen sample referenced to one of a negative control semen sample, **c**) Post-treatment TUNEL positivity% in semen sample referenced to one of a negative control semen sample

**Fig. 4:**
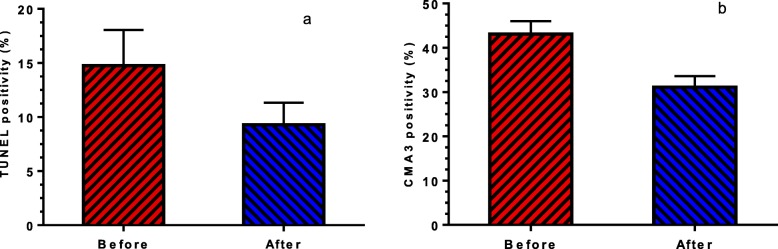
Comparison **a**) The mean sperm DNA fragmentation percentage (TUNEL positivity%, *P* = 0.0401) and **b**) The mean sperm protamine deficiency (CMA3 positivity%, *P* = 0.0001), before and after letrozole treatment

Figure 3 compares flow cytometry analysis of sperm TUNEL positivity percentage obtained from iOAT men with T:E_2_ ratio ≤ 10, before and after letrozole treatment. Also Fig. 4a indicates that the mean of sperm TUNEL positivity percentage decreased significantly (14.75 ± 3.29 versus 9.28 ± 2.04; *P* = 0.0401) after 3 months of letrozole administration.

The mean of sperm CMA3 positivity percentage decreased significantly (43.12 ± 2.91 versus 31.12 ± 2.46; *P* = 0.0001) after treatment (Fig. 4b). Moreover, there is a significant correlation between the percentage of sperm CMA3 positivity and sperm DNA fragmentation (r = + 0.832, *P* < 0.001).

**Fig. 5:**
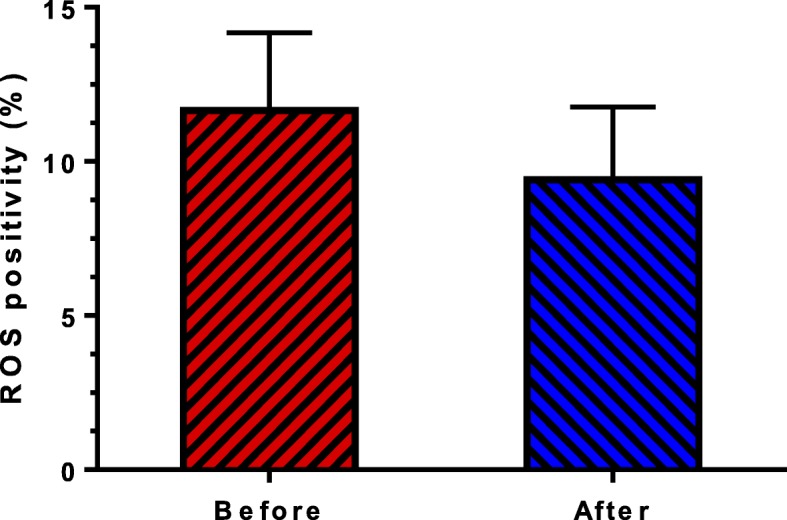
Comparison the mean percentage sperm positive for ROS before and after letrozole treatment (*P* = 0.2616)

Figure 5 indicates the mean sperm DCF positivity reduced after letrozole treatment (11.66 ± 2.52 versus 9.4 ± 2.36; *P* = 0.261), but this reduction was not statistically significant.

## Discussion

The results of this study showed that letrozole increases the serum testosterone levels and decreases the serum estradiol levels in iOAT individuals with T:E_2_ ratio ≤ 10, significantly, (Fig. 1a-b). It is interesting to note that the T:E_2_ ratio increased to higher than 10 (Fig. 1c) and this ratio improved 1600% after letrozole treatment. These results are consistent with the previous reports [15–19].

In men, the main source of estrogen is from the conversion of testosterone to estradiol catalyzed by the aromatase enzyme in the endoplasmic reticulum of testicular Leydig cells [41]. Estrogen has a strong negative feedback on the hypothalamus pituitary axis and can decrease the production and release of FSH and LH [24]. In the male reproductive system, inhibition of estrogen production by aromatase inhibitor, can increase FSH and LH levels, which, in turn, increase the intratesticular testosterone level and improve spermatogenesis [23]. Therefore, the hormonal balance created following letrozole administration, may be related to this inhibitory effect. This effect is probably due to aromatase inhibition in the testicular Leydig cells, which limits estrogen production, and thereby maintains testosterone level [42]. In addition, estrogen has direct and adverse effects on testicular germinal epithelium [43]; hence, the low T:E2 ratio may be responsible for impaired spermatogenesis, which can reverse by letrozole. One of the shortcomings of this study is that FSH and LH were not measured in parallel with estrogen and testosterone hormones.

Our results showed that sperm concentration, sperm motility and sperm morphology improved 260, 61 and 240%, respectively after letrozole treatment (Fig. 2a-c). These results are consistent with Previous studies [15–19]. This improvement in sperm parameters is due to the hormonal balance induced by letrozole through the inhibition of estradiol and increase of FSH and testosterone levels as markers of spermatogenesis and Sertoli cell function.

In Fig. 5 it was indicated that the degree of ROS reduced after letrozole treatment, but this reduction was not statistically significant. This is probably related to our inclusion criteria, because individuals at high risk of oxidative stress were not included in our study. Previous studies have shown that ROS levels in infertile men are higher than normal men [44]. ROS negatively affects the hypothalamus pituitary axis and decreases LH and FSH secretion, which, in turn, decrease the intratesticular testosterone level [45]. On the other hand, ROS can raise aromatase activity, which results in more E_2_ production and the inhibition of testosterone release [45]. It seems that, increased E_2_ in iOAT patients with T:E_2_ ratio ≤ 10 is likely to be related to high ROS level. Nonetheless, further research is required to evaluate this hypothesis. It is also important to note that, testosterone has antioxidant activity [45], and high level of estrogen can, inversely, suppress the expression of antioxidant enzyme [46]. Therefore, decrease of ROS level in our study may be related to the hormonal balance created in patients.

One of the important and essential elements involved in proper sperm formation during spermiogenesis is the proper replacement of histone with protamine [47–49]. The results of our study showed that the degree of protamine deficiency, assessed by CMA3 staining, decreased significantly after letrozole administration, (Fig. 4b). Researches have shown that protamine deficiency makes the sperm prone to DNA fragmentation [47–49]. Consequently, we also assessed DNA integrity through the TUNEL assay. The results of this assessment revealed that the degree of DNA fragmentation was lower after letrozole administration (Fig. 4a).

Three mechanisms that account for DNA fragmentation include: 1) defective chromatin packaging due to endogenous nuclease (topoisomerase II) inactivity to re-ligate the nicks during spermiogenesis, 2) abortive apoptosis and 3) DNA damage due to oxidative stress during the epididymal passage when mitochondria becomes active for ATP production during maturation in epididymis [50–53] . Considering that, we did not observed a significant decrease in ROS production, the improvement of DNA fragmentation is probably related to improved chromatin packaging and apoptotic pathway,which are mainly dependent on the levels of sex hormones [54]. Indeed, several studies have revealed that hormonal imbalance, altered FSH and LH levels, can result in DNA damage. These studies have also reported that the administration of FSH can reduce DNA damage in men with iOAT [55, 56]. The FSH mechanism of action in reducing DNA damage, probably correlates with its anti-apoptotic and maturation promoting effects on Sertoli and germ cells. So, suppressing FSH can increase apoptotic DNA fragmentation [55, 57]. In addition, the role of FSH in sperm maturation is related to the replacement of histones with protamines during spermiogenesis [56]. Therefore, it seems that letrozole along with the increase of FSH could improve chromatin integrity through the proper replacement of histones with protamines. Similar to previous studies [52, 58], we observed a strong correlation between the percentage of sperm CMA3 positivity and DNA fragmentation (sperm TUNEL positivity). Such a degree of strong correlation in this study (r = + 0.832, *P* < 0.001) was probably related to the inclusion of a specific population of men with iOAT in this study.

A notable result of this study was achieving four spontaneous pregnancies (20%) following letrozole treatment, which was consistent with the previous report [19].

In parallel with aromatase efficiency, letrozole efficiency was believed to be affected by numerous factors, including aromatase polymorphisms [59] and BMI [41]. The former was not considered in our study, but the comparison of pre- and post-treatment values of BMI and its correlation with letrozole efficiency in improving sperm parameters and hormonal balance were investigated. There was no significant difference in the BMI value before and after the letrozole treatment. Also, no significant correlation was observed between the baseline BMI and improvement in sperm parameters and hormonal balance following letrozole administration. Similar to previous studies [18, 41], our results showed that baseline BMI cannot predict the results of letrozole administration in improvement of spermatogenesis.

In our study, only five patients reported decreased libido during the treatment. This was probably related to lower estrogen levels in these individuals. Appropriate levels of testosterone and estradiol and their proper ratio are required for libido [60]. The effect of estradiol on libido is related to testosterone levels. Studies have shown that in men with low testosterone levels, estradiol administration can increase sexual desire. Reversely, libido decreases after the estradiol treatment in men with normal testosterone levels [61–63]. Therefore, it seems that letrozole administration in iOAT men with low testosterone levels can decrease libido through the reduction of estradiol.

One of the limitations of this study, similar to other research in this field [16, 18, 19], was the low number of patients studied, due to the low number of eligible patients to participate in this research. Also, the lack of control group in this research is due to the unwillingness of normal men to participate in research, which is one of the main challenges of human studies.

## Conclusion

The results of this study showed that letrozole treatment in iOAT patients with T:E_2_ ratio ≤ 10 can increase testosterone level, decrease estradiol level and improve T:E_2_ ratio (1600%) as well as hormonal balance, which regulate male reproductive functions. In addition, sperm concentration, sperm motility and sperm morphology improved 260, 61 and 240%, respectively. Furthermore, this study showed, for the first time, the degrees of protamine deficiency and DNA fragmentation were significantly reduced after treatment with letrozole. Finally, a notable result of this study was achieving four spontaneous pregnancies (20%) following letrozole treatment. Based on the results of this study, letrozole treatment can be considered as an effective treatment for iOAT patients with T:E_2_ ratio ≤ 10.

## Data Availability

The primary data for this study is available from the authors upon direct request.

## Abbreviations

BMI: Body mass index
iOAT: Oligo/astheno/teratozoospermia
T: Testosterone
E_2_: Estradiol
TUNEL: Terminal deoxynucleotidyl transferase-mediated fluorescein-dUTP nick end labeling
CMA3: Chromomycin A3
ROS: Reactive oxygen species
DCFH-DA: 2′, 7′-dichlorodihydrofluorescein diacetate
FSH: Follicle-stimulating hormone
LH: Luteinizing hormone
WHO: World Health Organization
TdT: Terminal deoxynucleotidyl transferase
SEM: Standard Error of Mean
